# Network Pharmacology and Experimental Assessment to Explore the Pharmacological Mechanism of Qimai Feiluoping Decoction Against Pulmonary Fibrosis

**DOI:** 10.3389/fphar.2021.770197

**Published:** 2021-12-03

**Authors:** Yingying Yang, Lu Ding, Tingting Bao, Yaxin Li, Jing Ma, Qingwei Li, Zezheng Gao, Siyu Song, Jing Wang, Jiachao Zhao, Ziyuan Wang, Daqing Zhao, Xiangyan Li, Zeyu Wang, Linhua Zhao, Xiaolin Tong

**Affiliations:** ^1^ Graduate College, Beijing University of Chinese Medicine, Beijing, China; ^2^ Institute of Metabolic Diseases, Guang’anmen Hospital, China Academy of Chinese Medical Sciences, Beijing, China; ^3^ Jilin Ginseng Academy, Key Laboratory of Active Substances and Biological Mechanisms of Ginseng Efficacy, Ministry of Education, Jilin Provincial Key Laboratory of Bio-Macromolecules of Chinese Medicine, Changchun University of Chinese Medicine, Changchun, China; ^4^ College of Integrated Traditional Chinese and Western Medicine, Changchun University of Chinese Medicine, Changchun, China; ^5^ Affiliated Hospital of Changchun University of Chinese Medicine, Changchun, China; ^6^ Department of Scientific Research, Changchun University of Chinese Medicine, Changchun, China

**Keywords:** COVID-19, pulmonary fibrosis, QM formula, network pharmacology, epithelial–mesenchymal transition, extracellular matrix accumulation, TGF-β1/Smad3 pathway

## Abstract

Pulmonary fibrosis (PF) is one of the pathologic changes in COVID-19 patients in convalescence, and it is also a potential long-term sequela in severe COVID-19 patients. Qimai Feiluoping decoction (QM) is a traditional Chinese medicine formula recommended in the Chinese national medical program for COVID-19 convalescent patients, and PF is one of its indications. Through clinical observation, QM was found to improve the clinical symptoms and pulmonary function and reduce the degree of PF of COVID-19 convalescent patients. To further explore the pharmacological mechanisms and possible active components of QM in anti-PF effect, UHPLC/Q-TOF-MS was used to analyze the composition of the QM extract and the active components that can be absorbed into the blood, leading to the identification of 56 chemical compounds and 10 active components. Then, network pharmacology was used to predict the potential mechanisms and targets of QM; it predicted that QM exerts its anti-PF effects *via* the regulation of the epithelial–mesenchymal transition (EMT), extracellular matrix (ECM) degradation, and TGF-β signaling pathway. Finally, TGF-β1–induced A549 cells were used to verify and explore the pharmacological effects of QM and found that QM could inhibit the proliferation of TGF-β1–induced A549 cells, attenuate EMT, and promote ECM degradation by inhibiting the TGF-β/Smad3 pathway.

## Introduction

Many interstitial lung diseases, such as idiopathic interstitial pneumonias and acute lung injury, can progress to pulmonary fibrosis (PF). Idiopathic PF (IPF) is a devastating, irreversible, and chronic progressive form of PF, and lung transplantation is the only treatment that can change the outcome of IPF patients (Richeldi et al., 2017). There are three million IPF patients around the world, and its incidence increases dramatically with age (Martinez et al., 2017). PF is also a pathological phenomenon in the development of various respiratory diseases, such as viral pneumonia and chronic obstructive pulmonary disease (COPD) (Meyer, 2017). The Coronavirus disease 2019 (COVID-19), which is caused by severe acute respiratory syndrome coronavirus 2 (SARS-CoV-2) (Asselah et al., 2021), has become a worldwide pandemic, infecting over 222 million people and killing more than 4.9 million people as of October 2021. However, the absence of SARS-CoV-2 nucleic acid in COVID-19 patients does not guarantee complete recovery, especially in severe and critical patients. A series of problems can occur during the COVID-19 recovery period, called post-acute COVID-19 syndrome, involving multiple disorders of the cardiovascular system, respiratory system, endocrine system, nervous system, urinary system, digestive system, and skin (Nalbandian et al., 2021). Several clinical studies have found that patients during the COVID-19 recovery period have imaging signs of PF, especially in critical patients. For example, a Chinese cohort study about post–COVID-19 indicated that in approximately 50% of 349 patients, at least one abnormal pattern was observed in high-resolution computed tomography (CT) scans of the chest at 6 months after discharge, with most abnormalities observed by CT being ground-glass opacities (Huang et al., 2021). Moreover, pathological studies of patients who died of COVID-19 revealed that PF was widespread in lung tissues (Bian, 2020). Consequently, PF could be a serious consequence of SARS-CoV-2 infection, according to the current information on COVID-19 and the data from severe acute respiratory syndrome (SARS) and Middle East respiratory syndrome (MERS) (George et al., 2020). Additionally, the major risk factors for severe COVID-19 are shared with that of IPF (George et al., 2020), including male sex, age, hypertension, diabetes, and other comorbidities. However, the treatment of IPF also remains supportive presently, with pirfenidone and nintedanib being among the few drugs that have anti-PF effects (King et al., 2014; Flaherty et al., 2019). Therefore, there is an urgent need to develop novel therapeutic strategies to delay the progressive stages of PF and deal with the possible arrival of post-COVID-19–related PF.

During the process of PF, epithelial cells can respond to microenvironmental cues and convert to mesenchymal cells, such as fibroblasts and myofibroblasts, which are direct effectors of organ fibrosis, *via* a process called the epithelial–mesenchymal transition (EMT) (King et al., 2011; Salton et al., 2019). Transforming growth factor beta (TGF-β) is an important fibrotic cytokine that can induce EMT and cause fibroblast differentiation into myofibroblast (Yan et al., 2014). Upon stimulation with cytokines such as TGF-β, angiotensin II, and interleukin-6 (IL-6), mesenchymal cells, especially myofibroblasts, could secrete large amounts of extracellular matrix (ECM), such as collagen I, laminin (LN), fibronectin (FN), and alpha-smooth muscle actin (α-SMA) (Meng et al., 2016; Forcina et al., 2019; Ji et al., 2019). Excessive deposition of ECM could result in scarring and destruction of the lung architecture (Bonnans et al., 2014). Thus, the inhibition of TGF-β–mediated EMT and ECM accumulation is a potential therapeutic strategy to prevent PF.

Based on the traditional Chinese medicine (TCM) theory, Qi-Yin deficiency and phlegm-stasis in channels are the key factors in the pathogenesis of PF; they are also observed in many COVID-19 convalescent patients, especially those with PF. Qimai Feiluoping decoction (QM), a TCM formula prescribed by Professor Tong Xiaolin, could nourish Qi-Yin, dissipate phlegm, invigorate blood circulation, and dredge collaterals. Therefore, QM is used to treat the convalescent patients of COVID-19 with the syndromes of phlegm-stasis in channels*.* QM consists of 18 Chinese medicines (Table 1). Through clinical observation, QM was found to be effective in improving fatigue, asthma, respiratory function, and abnormal lung CT manifestations (ground-glass shadow, fiber rope shadow, and mesh shadow) in COVID-19 convalescent patients. QM is also recommended in the Chinese rehabilitation guideline for major dysfunctions of COVID-19 discharged patients (http://www.nhc.gov.cn/xcs/zhengcwj/202005/b15d59b5228341129cc8c5126f663b10.shtml) to treat COVID-19 convalescent patients. In addition, a randomized controlled clinical trial (RCT) is being performed in the Hubei Provincial Hospital of Traditional Chinese Medicine (Wuhan, China) to further clarify i) the clinical effects of QM against COVID-19–related PF and ii) its stimulatory effects on lung function improvement in severe and critical COVID-19 convalescent patients (registration number: ChiCTR2000032165). The preliminary statistical results of the RCT have indicated the effectiveness of QM for preventing post-COVID-19–related PF.

**TABLE 1:** Compositions of QM.

Chinese name	Taxonomy name	Drug name	Abbr.	Family	Weight (g)	Part used	Voucher specimen
Huang-qi	*Astragalus mongholicus* Bunge	Astragali radix	HQ	Fabaceae	15	Root	202106-01
Dang-shen	*Codonopsis pilosula* (Franch.) Nannf.	Codonopsis radix	DGS	Campanulaceae	9	Root	202106-02
Bai-zhu	*Atractylodes macrocephala* Koidz.	Atractylodis macrocephalae rhizoma	BZ	Asteraceae	9	Rhizome	202106-03
Nan-sha-shen	*Adenophora triphylla* (Thunb.) ADC.	Adenophorae radix	NSS	Campanulaceae	9	Root	202106-04
Bei-sha-shen	*Glehnia littoralis* (A. Gray) F. Schmidt ex Miq.	Glehniae radix	BSS	Apiaceae	9	Root	202106-05
Mai-dong	*Ophiopogon japonicus* (Thunb.) Ker Gawl.	Ophiopogonis radix	MD	Asparagaceae	15	Rhizome	202106-06
Chen-pi	*Citrus x aurantium* L.	Citri reticulatae pericarpium	CP	Rutaceae	9	Rind	202106-07
Fu-ling	*Poria cocos* (Schw.) Wolf.	Poria	FL	Polyporaceae	15	Sclerotium	202106-08
Ban-xia	*Pinellia ternata* (Thunb.) Makino	Pinelliae rhizoma	BX	Araceae	6	Tuber	202106-09
Dan-shen	*Salvia miltiorrhiza* Bunge	Salviae miltiorrhizae radix et rhizoma	DS	Lamiaceae	9	Root	202106-10
Zhe-bei-mu	*Fritillaria thunbergii* Miq	Fritillariae thunbergii bulbus	ZBM	Liliaceae	3	Bulb	202106-11
Shui-zhi	*Whitmania pigra* Whitman	Hirudo	SZ	Hirudinidae	3	Whole animal	202106-12
Tu-bie-chong	*Eupolyphaga sinensis* Walker	Eupolyphaga steleophaga	TBC	Corydiidae	3	Whole animal	202106-13
Gan-cao	*Glycyrrhiza glabra* L.	Glycyrrhizae radix et rhizoma	GAC	Fabaceae	6	Rhizome	202106-14
Shan-zha	*Crataegus pinnatifida* Bunge	Crataegi fructus	SHZ	Rosaceae	3	Fruit	202106-15
Shen-qu		Massa medicata fermentata	SQ		3	Leavening	202106-16
Mai-ya	*Hordeum vulgare* L.	Hordei fructus germinatus	MY	Poaceae	3	Fruit	202106-17
Shan-yao	*Dioscorea oppositifolia* L.	Dioscoreae rhizoma	SY	Dioscoreaceae	9	Rhizome	202106-18

Network pharmacology, as a systematic pharmacology research method, shares much with the key ideas of the holistic view of TCM. Based on network pharmacology, the mechanisms and possible active ingredients of many TCM formulas (Qing-Luo-Yin, Liu-Wei-Di-Huang pill, and Shen-Qi-Di-Huang decoction) against complex diseases, such as cancer, metabolic syndrome, and diabetic nephropathy, were revealed (Li et al., 2007; Li and Zhang, 2013; Di et al., 2018). A previous network pharmacological analysis had predicted the mechanism underlying the protective effects of QM against PF: VEGF, TNF-α, IL-6, MMP9, and TGF-β1 are potential targets, and the activation of VEGF, Toll-like receptor 4, MAPK, and TGF-β1 signaling pathways may protect patients against PF (Jin et al., 2021). However, the active components of QM were not identified, and these underlying mechanisms have not been further confirmed. Therefore, we used UHPLC/Q-TOF-MS analysis to identify the compounds of QM and combined network pharmacology to predict the potential mechanisms underlying the anti-PF effects of QM. TGF-β1–induced A549 cells were used to verify the hypothesis from the network pharmacological analysis. This study provides new insights into the pharmacological mechanism and possible active components of QM, which could support its clinical application to prevent progressive fibrosis in patients with pulmonary diseases.

## Materials and Methods

### Preparation of the QM Extract

The 18 Chinese medicines of QM (Table 1) were purchased from Beijing General Pharmaceutical Corporation (Beijing, China) and provided by the Department of Pharmacy, Affiliated Hospital of Changchun University of Traditional Chinese Medicine (Changchun, China). The voucher specimens were deposited at the Jilin Ginseng Academy, Changchun University of Chinese Medicine (Changchun, China). According to the standard procedure from Chinese Pharmacopoeia (2020 edition), all of the raw materials (138 g, clinical dosage) were decocted by distilled water twice (1 h each time) at a mass ratio of 1:10 to obtain the water extract of QM. The water extract was filtered and centrifuged to obtain the supernatant, which was dried in vacuum to obtain the powdery extract with a yield of 24.6% (34 g). The QM powdery extract was used for the UHPLC/Q-TOF-MS analysis and further experiments (Zhang et al., 2020).

### UHPLC/Q-TOF-MS Analysis of the QM Extract

The following reference compounds were used for the UHPLC/Q-TOF-MS analysis: calycosin 7-O-glucoside (batch number: 5240), complanatuside (batch number: 3037), ononin (batch number: 3811), neoisoliquiritin (batch number: 6971), neoliquiritin (batch number: 7543), formononetin (batch number: 3523), quercetin (batch number: 1116), genistin (batch number: 9435), oleanolic acid (batch number: 1339), oroxylin A (batch number: 6180), isoquercitrin (batch number: 5650), atractylodin A (batch number: 7361), and methylnissolin-3-O-glucoside (batch number: 3816). All were bought from Shanghai Sidande Standard Technical Service Co., Ltd. Shanghai, China. Schaftoside (batch number: DST200524-006), calycosin (batch number: DST190915-012), isoliquiritigenin (batch number: DST190719-016), naringin (batch number: DST191011-099), naringenin (batch number: DST200109-100), peimine (batch number: DST200109-011), peiminine (batch number: DSTDB001201), hesperidin (batch number: DST190716-038), neohesperidin (batch number: DSTDX003901), nobiletin (batch number: DSTDC003701), lobetyolin (batch number: DATDD003501), caffeic acid (batch number: DST190517-013), 5-O-demethylnobiletin (batch number: DSTDQ009401), 3′-demethylnobiletin (batch number: DST210212-120), betulinic acid (batch number: DST190508-026), and isoastragaloside I (batch number: DST180315-022) were purchased from Chengdu Deste Biotechnology Co., Ltd. (Chengdu, China). Liquiritin (batch number: PS012028) was purchased from Chengdu Pusi Biotechnology Co., Ltd. (Chengdu, China). Rapid characterization of the multicomponent from the QM extract was performed using a Waters ACQUITY UPLC I-Class Plus/Xevo G2-XS QTOF system (Waters, Milford, United States). Chromatographic separation was performed on a Waters ACQUITY UPLC BEH C18 column (2.1 mm × 100 mm, 1.7 µm) maintained at 30°C. The mobile phase was composed of 0.1% formic acid in water (A) and 0.1% formic acid in acetonitrile (B) running at 0.3 ml/min consistent with the following optimal gradient elution program: 0–4 min, 2–8% B; 4–7 min, 8–15% B; 7–11 min, 15–21% B; 11–14 min, 21% B; 14–16 min, 21–32% B; 16–19 min, 32% B; 19–24 min, 32–50% B; 24–27 min, 50–80% B; and 27–30 min, 80–95% B. The injection volume was 1 μl. High-resolution MS data were recorded on a Xevo G2-XS QTOF mass spectrometer by MS^E^ in both the positive and negative ESI modes. The source parameters were as follows: capillary voltage, –2.0 kV in the negative ESI mode and 3.0 kV in the positive ESI mode; source temperature, 100°C; and cone voltage, 40 V. The mass analyzer scanned over the mass range of *m/z* 50–1,500 at a low collision energy of 6 V and a high collision energy of 40–60 V for the recording of MS^1^ and MS^2^ spectra by MS^E^, respectively. Processing of the obtained MS^E^ data was conducted using UNIFI^TM^ 1.9.3.0 software (Waters, Milford, United States).

### Characterization of QM Components From Rat Plasma

Seven male Wistar rats (weighing 250 ± 20 g) were treated by oral gavage with the clinical equal dose of the QM powdery extract (3 g/kg/day, equal to 12.4 g raw materials/kg/day), and the blood samples were collected 10 days after oral administration of QM. Prior to analysis by UHPLC/IM-QTOF-MS, the plasma samples were processed as described below. An aliquot of 200 μl of the plasma sample was added with 600 μl of methanol. The mixed sample was then vortexed for 3 min and centrifuged at 13,000 rpm for 10 min, to precipitate the proteins. The resulting supernatant was evaporated to dryness under a stream of nitrogen at 40°C, and the dried residue was further dissolved in 200 μl of methanol–water (1:1, *v*/*v*) solution. After centrifugation at 13,000 rpm for 10 min, the supernatant was used as the test solution and injected for analysis. Characterization of the QM components from the rat plasma was performed using a Waters ACQUITY UPLC I-Class/Vion IMS-QTOF mass spectrometer (Waters, Manchester, United Kingdom) by MS^E^ in both positive and negative ESI modes, following the same chromatography and MS conditions as described above for characterizing the multiple components from the QM extract.

### Collection of Components and Disease Targets

The PubChem database was used to obtain the molecular structure (SDF format) of each QM compound (Wang et al., 2012). Then, the molecular structural files of identified compounds were uploaded to the PharmaMapper database, the ChemMapper database, and the SwissTarget database to predict their potential targets (Gong et al., 2013; Gfeller et al., 2014; Wang et al., 2017). Meanwhile, PF-related targets were collected using the GeneCards database (Stelzer et al., 2016). The gene and UniProt IDs of these potential targets were obtained from the UniProt database. Finally, the overlapping targets between the potential therapeutic targets of QM and the disease targets in PF were obtained for further network analysis.

### Network Construction and Analysis

Cytoscape 3.8.0 was used to visualize the relationship between components of QM and the potential targets of each compound. To explore the interaction among target proteins, overlapping targets were uploaded to STRING and the information about the protein–protein interaction (PPI) was obtained(Szklarczyk et al., 2015). Importantly, the significant Gene Ontology (GO), KEGG, and Reactome pathways were screened by the DAVID database (Huang et al., 2009).

### Molecular Docking

The molecular structures of the compounds were searched from the PubChem database and reconstructed in the ChemBioDraw. Then, these structures were saved as “mol2” file with the energy minimized and saved as docking ligands as “pdbqt” files in AutoDock version 4.2, respectively. The PDB database (https://www.rcsb.org/) was used to retrieve the 3D structure of TGF-β1 (PDB ID: 1KLA) and TGF-β receptor 1 (TGF-βR1, PDB ID: 1PY5) with the files in the “pdb” format. Discovery Studio Client version 4.5 was used to hydrogenate proteins, remove water, and remove ligand molecules. AutoDock version 4.2 was used to add the nonpolar hydrogen, calculate Gasteiger charges for the protein, save it as “pdbqt” files, and finally, run Vina for molecular docking. The active site of molecular docking was determined by the ligand coordinates in the target protein complex. The ligand was set to be flexible, and the receptor was set to be rigid. Each receptor–ligand interaction generates 50 conformations, and the conformation with the best affinity was used as the final docking conformation. If the binding energy is less than 0, the compound (ligand) and protein (receptor) can bind spontaneously. If binding energy ≤ −1.2 kCal/mol, the ligand–receptor was considered stable (Gomez-Garcia et al., 2018).

### Cell Culture and Model Establishment

Human adenocarcinomic A549 cells with type II alveolar epithelial cell features were bought from the cell bank of the Shanghai Institute of Cell Biology, Chinese Academy, and cultured in RPMI 1640 containing 10% fetal bovine serum (FBS, Clark Bioscience, Claymont, United States), 100 kU/L penicillin, and 100 mg/L streptomycin (Biosharp, Hefei, China) at 37°C in a 5% CO_2_ humidified incubator. When the cells were in good condition, TGF-β1 (5 ng/ml) was added to induce A549 cell transformation into mesenchymal-like cells. After 24 h, the A549 cells were observed under the microscope (Olympus CKX53, Japan) to see whether their morphology had changed from hexagonal to a spindle shape after TGF-β1 treatment (Zhang et al., 2019).

### Cell Viability Assay

A549 cells were seeded into 96-well plates at a density of 3,000, 6,000, or 8,000 cells/well. The effect of QM on cell viability was analyzed after treatment for 24, 48, or 72 h at a concentration of 7.8–1,000 μg/ml. Additionally, A549 cells were divided into the control group (dimethyl sulfoxide, DMSO alone), the model group (5 ng/ml TGF-β1), and the treatment group (5 ng/ml TGF-β1 + 7.8–1,000 μg/ml QM) to examine the effect of QM on the viability of TGF-β1–treated cells. After treatment for 24, 48, or 72 h, MTT (0.5 mg/ml) was added (Solibol, Beijing, China). DMSO (150 μl) was used to dissolve formazan crystals. Absorbance was measured at 490 nm using a microplate reader. The cell survival rate was calculated as the percentage of each group relative to the control group.

### Quantitative Real-Time PCR Analysis

Total RNA was extracted from A549 cells using a total RNA extraction kit. Next, 1 μg of total RNA was reverse transcribed into cDNA with the iScript cDNA synthesis kit. A Bio-Rad CFX96 system was used to perform quantitative real-time PCR (qPCR) analysis, and the relative mRNA levels were calculated using the 2^−ΔΔCt^ method using GAPDH for normalization (Zhang et al., 2020). The primer sequences to amplify the genes encoding N-cadherin, E-cadherin, vimentin, α-SMA, LN, FN, collagen I, TGF-βR1, Smad7, and GAPDH are listed in Supplementary Table S2.

### Western Blot Analysis

Antibodies against LN (ab11575), FN (ab2413), collagen I (ab34710), α-SMA (ab5694), E-cadherin (ab40772), N-cadherin (ab76011), vimentin (ab8978), TGF-βR1 (ab31013), Smad3 (ab40854), p-Smad3 (S423/S425 and ab52903), Smad7 (ab216428), and GAPDH (ab8245) were purchased from Abcam (Cambridge, MA, United States). After QM treatment, the total protein fraction was extracted from A549 cells by lysis in ice-cold RIPA buffer. Proteins were separated by SDS-PAGE and transferred onto PVDF membranes. After blocking for 1 h at room temperature with a blocking buffer containing 5% BSA, the membranes were incubated with the primary antibody overnight at 4°C. After washing, the membranes were incubated with the HRP-conjugated secondary antibody for 2 h. The protein bands were visualized using a chemiluminescent imaging system (ChemiDoc XRS+, Bio-Rad) and quantified by ImageJ software (Jiang et al., 2021).

### Statistical Analysis

The data are expressed as mean ± standard deviation from three independent experiments. All of the data were statistically analyzed using GraphPad prism 9.0. Multiple groups were compared by one-way ANOVA (Tukey’s *post hoc* test) to determine statistical significance. For all statistical analyses, *p* < 0.05 was considered to be statistically significant.

## Results

### Chemical Components and Quality Control of QM Extract

**FIGURE 1:**
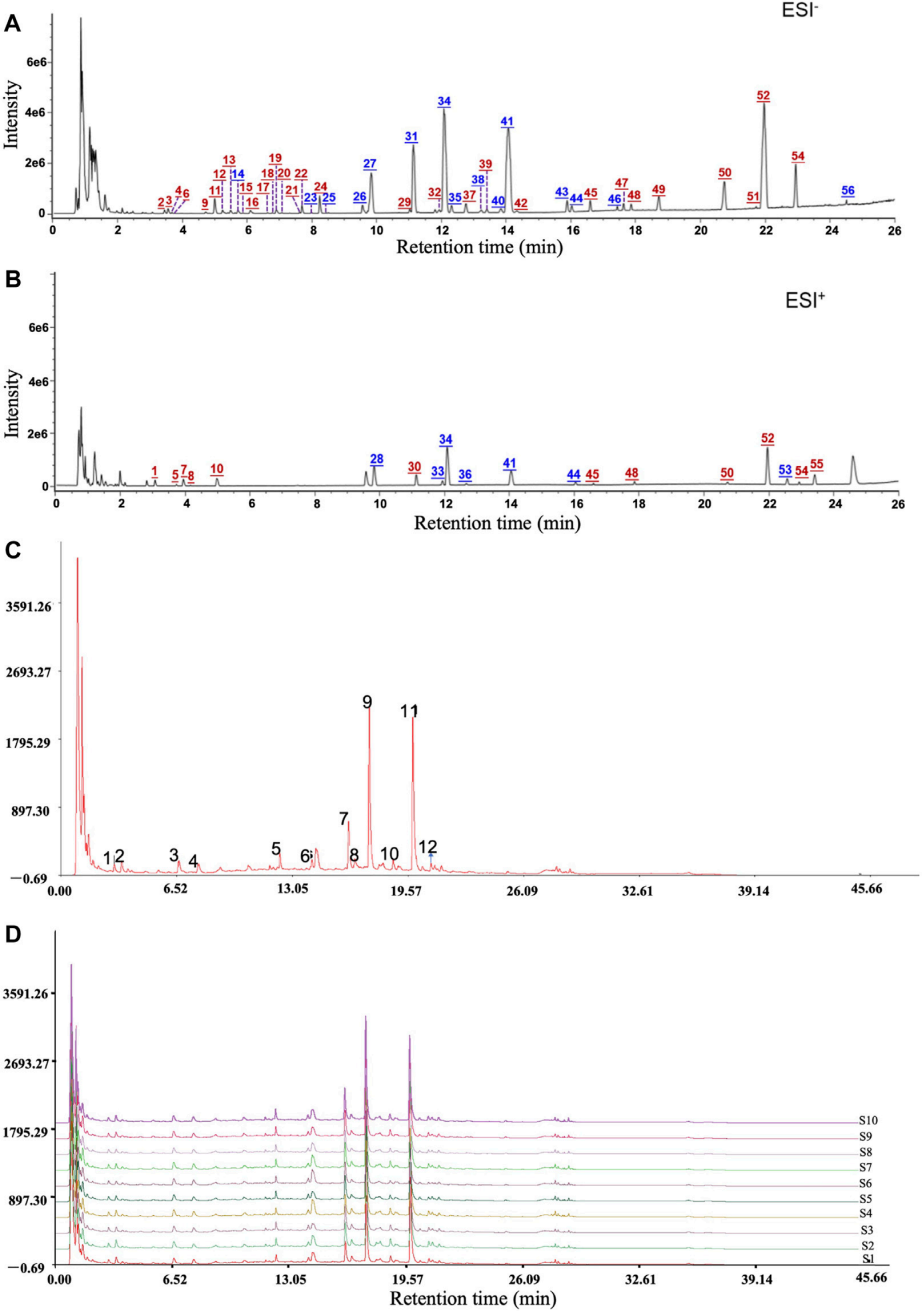
Mass spectrum chromatograms of QM to screen for active components and analyze the reproducibility. **(A)** Negative ion mode. **(B)** Positive ion mode. Blue numbers represent those compounds that were identified through comparison with the reference standards. **(C,D)** The reproducible HPLC fingerprints of the mixture of 12 standard compounds and 10 batches of QM (S1–S10), using the Chinese Medicine Chromatographic Fingerprint Similarity Evaluation System (2012 Edition).

To identify the chemical components of QM and analyze the consistency between various batches of QM, UHPLC/Q-TOF-MS analysis was conducted. The extracted ion chromatograms of all the compounds are shown in Figures 1A,B. A total of 56 components were characterized, among which 19 were accurately identified *via* comparison with the reference compounds. Information of these characterized components, including their assignments to the single drugs, is presented in Supplementary Table S1. These components were mainly from nine botanical drugs, that is, astragali radix (Huang-qi), citri reticulatae pericarpium (Chen-pi), salviae miltiorrhizae radix et rhizoma (Dan-shen), glycyrrhizae radix et rhizoma (Gan-cao), glehniae radix (Bei-sha-shen), atractylodis macrocephalae rhizoma (Bai-zhu), pinelliae rhizoma (Ban-xia), codonopsis radix (Dang-shen), and fritillariae thunbergii bulbus (Zhe-bei-mu). Nineteen compounds were identified by comparison with reference compounds, namely, atractyloside A, complanatuside, schaftoside, calycosin 7-O-glucoside, liquiritin, isoliquiritigenin, naringin, peimine, hesperidin, neohesperidin, peiminine, lobetyolin, neoisoliquiritin, ononin, methylnissolin-3-O-glucoside, calycosin, naringenin, nobiletin, and isoastragaloside I. Moreover, 10 batches of QM samples were analyzed, and similarity values of 0.983–0.999 were obtained by comparing with the reference spectrum, which indicated that the QM formula was reproducible (Figures 1C,D).

### Network Establishment Between QM and PF

**FIGURE 2:**
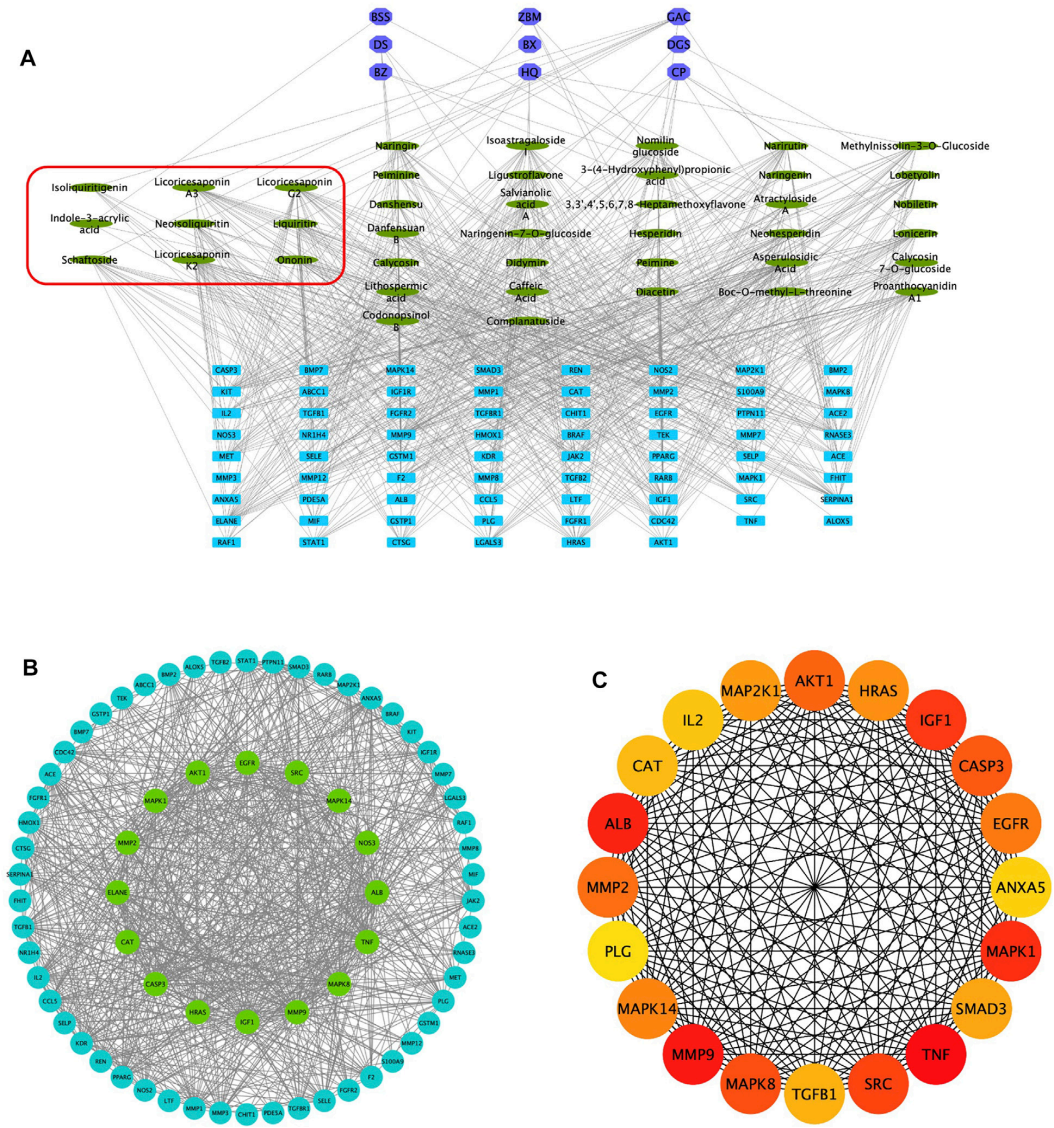
Potential targets of QM components were predicted by network pharmacology analysis. **(A)** Construction of the network of 43 components–70 overlapping targets–nine botanical drugs. The green ovals represent the 43 active ingredients, the purple octagons represent the nine Chinese medicines, and the blue rectangles represent the 70 targets. The components circled by the red box are active ingredients isolated from rats’ plasma. **(B)** Construction of the protein–protein network to identify potential targets of QM. The blue and green circles represent the overlapping targets. Topological analysis was used to screen more core targets of QM, according to degree ≥ 24, betweenness ≥ 0.01, and closeness ≥ 0.615, which are represented by the green circles. **(C)** The top 20 target networks are shown.

Combined with the PubChem database, 43 of the 56 chemical compounds from the UHPLC/Q-TOF-MS analysis had a well-defined molecular structure. Then, according to the “Network Pharmacology Evaluation Method Guidance” (Li, 2021), the 43 chemical compounds were used for the network pharmacological analysis. First, the PubChem database was used to obtain the molecular structure files of the 43 QM components. The potential therapeutic targets of the 43 ingredients were obtained from the PharmaMapper database, the ChemMapper database, the SwissTarget database, and the literature reports (Bunbupha et al., 2020; He et al., 2020a; Wang et al., 2021). These targets were screened according to many parameters, such as fit score ≥3.0 in the PharmaMapper database, score ≥0.4 in the ChemMapper database, and probability ≥0.4 in the SwissTarget database. After screening, we obtained the UniProt ID corresponding to each protein target and deleted reduplicated targets. Thus, a total of 452 potential therapeutic targets of QM were obtained (Supplementary Table S4). Second, the network of 43 active components and 452 potential targets of QM was constructed (Supplementary Figure S1). Furthermore, potential targets in PF were obtained from the GeneCards database, and 642 targets related to PF were obtained with the relevance score >11.63 (depending on the median to screen). Although the relevance score of angiotensin-converting enzyme 2 (ACE2) was lower than 11.63, ACE2 is an important target of COVID-19–related PF (Mourad and Levy, 2020), and QM is a TCM formula for the treatment of COVID-19 convalescent patients with PF. Therefore, ACE2 was also selected as a target of PF in this study, and a total of 643 targets of PF were screened out for further analysis. Based on 452 targets of QM and 643 targets in PF, 70 overlapping targets between QM and PF were obtained (Supplementary Table S3). Then, we established a network between the 43 QM components and 70 overlapping targets (Figure 2A). Through the network, we found that 2-succinyl-6-hydroxycyclohexa-2, 4-diene-1-carboxylic acid, and codonopsine were not connected with the overlapping targets. Then, the 70 overlapping targets were submitted to the STRING database to build the PPI network. As shown in Figure 2B, the PPI network had 70 nodes and 866 edges. Subsequently, topological screening with degree ≥24, betweenness ≥0.01, and closeness ≥0.615 was used to screen key targets (Figure 2B). In addition, the top 20 targets of QM, such as TNF, MMP9, ALB, MAPK1, IGF1, SRC, MAPK8, CASP3, AKT1, MMP2, EGFR, MAPK14, HRAS, MAP2K1, SMAD3, TGFB1, CAT, IL2, ANXA5, and PLG, were selected and ranked with the maximal clique centrality (MCC) score (Figure 2C). Taken together, 70 potential targets of the 43 components of QM were obtained by network pharmacology.

### Enrichment Analysis

**FIGURE 3:**
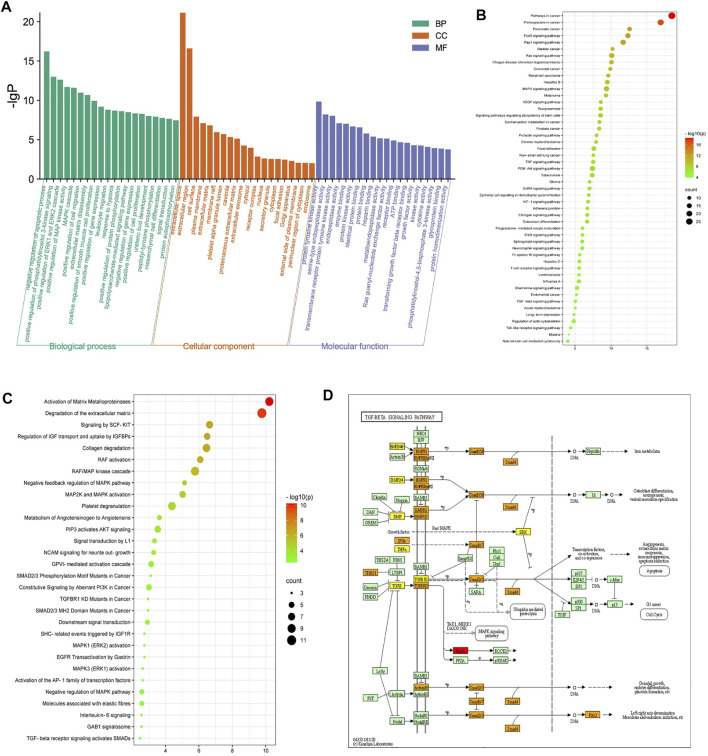
Enrichment analysis of the potential targets of QM against PF was conducted based on the DAVID database. **(A)** Top 20 biological process (BP) terms, cellular component (CC) terms, and molecular function (MF) terms are shown as a bar diagram, according to the -lg p value. **(B,C)** The top 50 entries of KEGG pathway analysis and the top 30 entries of the Reactome pathway analysis were selected and ordered according to the -lg p value. **(D)** The TGF-β signaling pathway modified from hsa04350. Red represents targets of QM, orange represents key targets participated in PF, and yellow represents overlapping targets between QM and PF.

The 70 overlapping targets were submitted to the DAVID database to conduct GO enrichment analysis, from which we obtained 402 biological process (BP) terms (Supplementary Table S5), 36 cellular component (CC) terms (Supplementary Table S6), and 77 molecular function (MF) terms (Supplementary Table S7). The top 20 entries were respectively selected from BP, CC, and MF, in order of -lg p value (Figure 3A). In the BP category, ECM disassembly, mesenchymal cell differentiation, EMT, the collagen catabolic process, and proteolysis were directly related to PF. In the MF category, the targets were mainly involved in protein tyrosine kinase activity, protein kinase activity, TGF-β receptor binding, type II TGF-β receptor binding, protease binding, fibroblast growth factor–activated receptor activity, and fibroblast growth factor binding. The DAVID database was also used to obtain 71 Reactome pathway terms and 99 KEGG pathway terms (Supplementary Tables S8, S9). The top 50 entries of the KEGG database (Figure 3B) and the top 30 entries of the Reactome database (Figure 3C) were selected depending on the -lg p value. The KEGG pathways mainly included FoxO, Rap1, MAPK, VEGF, and TGF-β signaling pathways. The Reactome pathways mainly included activation of matrix metalloproteinases, degradation of the ECM, signaling by SCF-KIT, regulation of IGF transport and uptake by IGFBPs, collagen degradation, and activation of Smads by TGF-β receptor signaling. Collectively, the network pharmacological analysis indicated that QM may inhibit the TGF-β signaling pathway to mediate ECM accumulation, fibroblast activation, and EMT to prevent the progression of PF. The key targets of the TGF-β signaling pathway that participated in PF are shown in Figure 3D.

### QM Inhibited TGF-β1–Induced A549 Cell Proliferation

**FIGURE 4:**
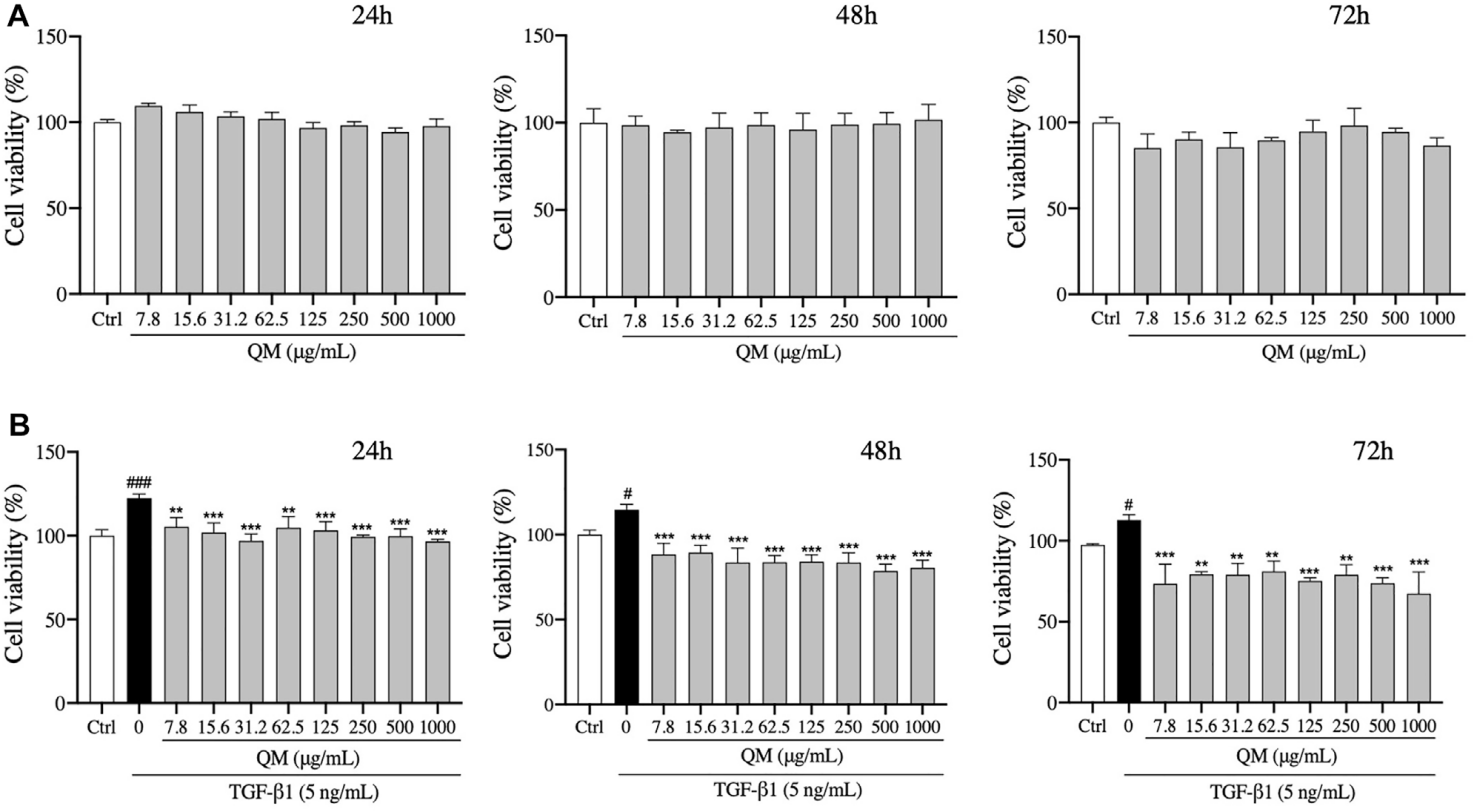
QM inhibited the proliferation of TGF-β1–stimulated A549 cells. **(A)** After treatment with different doses of QM for 24, 48, and 72 h, the cytotoxicity of QM in A549 cells was evaluated by the MTT assay. **(B)** In TGF-β1–induced A549 cells, the effect of different doses of QM on cell viability after treatment for 24, 48, and 72 h was evaluated by the MTT assay. Data are shown as mean ± standard deviation from three independent experiments. ^#^ *p* < 0.05, ^###^ *p* < 0.001 versus the control group; **p < 0.01 and ***p < 0.001 versus the model group (TGF-β1, 5 ng/ml).

The cytotoxicity of QM was tested. We found that QM at a concentration of 7.8–1,000 μg/ml was not cytotoxic for A549 cells after treatment for 24, 48, or 72 h (Figure 4A). Moreover, the viability of A549 cells was determined in the presence of TGF-β1 to mimic a pro-fibrotic environment. Compared with the control group, TGF-β1 significantly induced proliferation of A549 cells. After QM treatment at 7.8–1000 μg/ml for 24, 48, or 72 h, the proliferation of TGF-β1–induced A549 cells was decreased (Figure 4B). These data indicate that QM could inhibit the proliferation of TGF-β1–induced A549 cells.

### QM Attenuated EMT in TGF-β1–Induced A549 Cells

**FIGURE 5:**
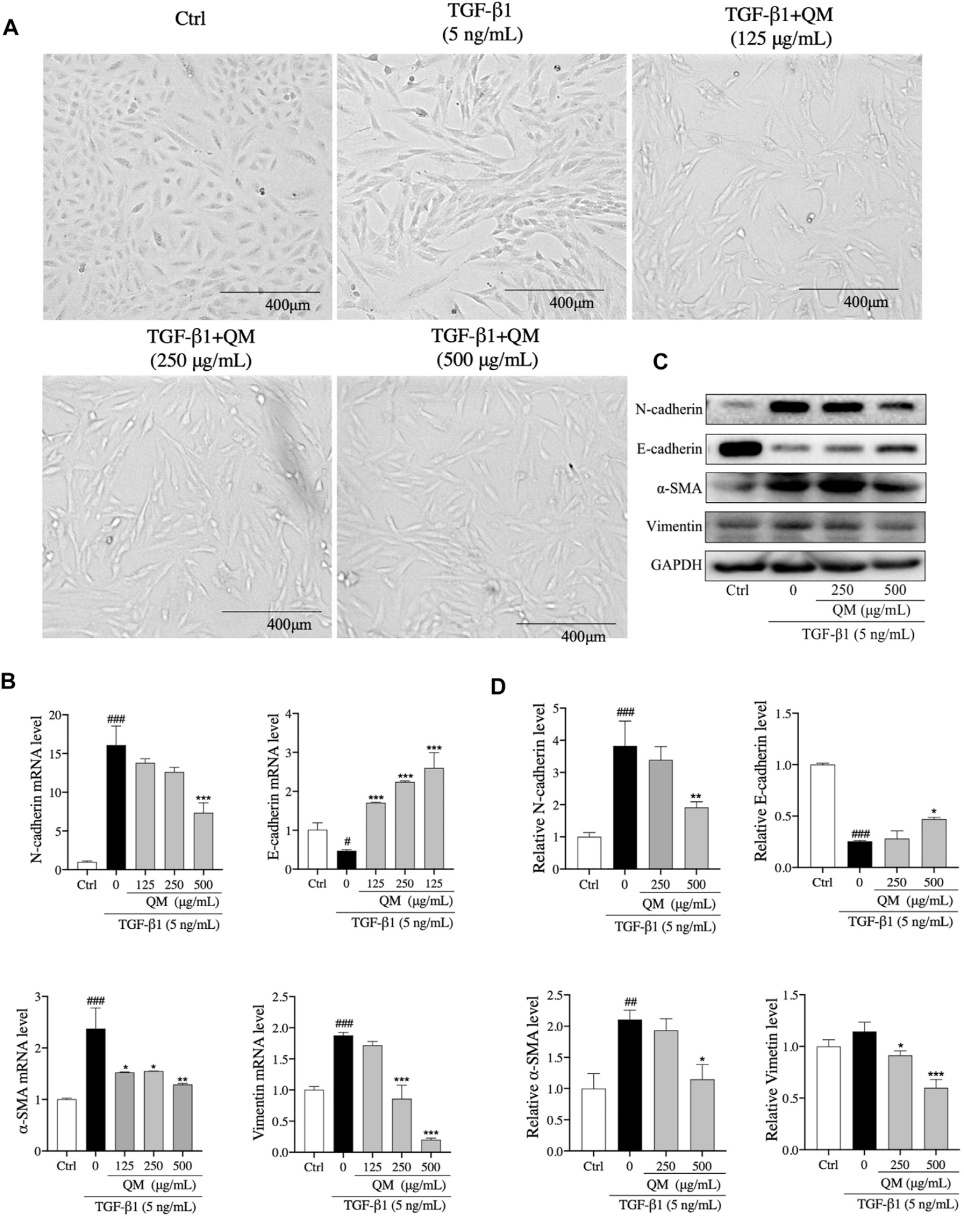
QM-attenuated TGF-β1–induced EMT in A549 cells. **(A)** Representative light microscopic images of A549 cells treated with TGF-β1 and/or QM (125, 250, or 500 μg/ml). **(B)** After incubation with QM and TGF-β1 for 72 h, the mRNA levels of N-cadherin, E-cadherin, α-SMA, and vimentin in A549 cells were determined by qPCR analysis. GAPDH was used for normalization. **(C,D)** After treatment for 72 h, the protein levels of N-cadherin, E-cadherin, vimentin, and α-SMA in A549 cells from the control, TGF-β1, and TGF-β1 + QM groups were determined by Western blot analysis. GAPDH was used as the loading control. The relative levels of these proteins were calculated based on gray values from three independent experiments and normalized with GAPDH. ^#^ *p* < 0.05, ^##^ *p* < 0.01, ^###^ *p* < 0.001 versus the control group; *p < 0.05, **p < 0.01, and ***p < 0.001 versus the model group (TGF-β1, 5 ng/ml).

TGF-β1 exerts strong pro-fibrotic effects and can induce EMT (Pezone et al., 2020), which is a phenomenon where inherent epithelial markers such as E-cadherin are lost and mesenchymal characteristics such as N-cadherin, vimentin, and α-SMA are gained (Cruz-Solbes and Youker, 2017). The morphology of A549 cells changes from hexagon to an elongated spindle-like shape when EMT occurs (Zhang et al., 2019). In the present study, after treatment with TGF-β1 (5 ng/ml) for 72 h, a spindle-like shape of A549 cells was observed (Figure 5A). When A549 cells were treated with QM at 125, 250, or 500 μg/ml, the mesenchymal-like morphology was attenuated compared with the TGF-β1–induced group (Figure 5A). To further verify the effects of QM in inhibiting EMT at the molecular level, qPCR and Western blot analyses were used to detect the EMT-related markers. After 72 h of incubation, qPCR results showed that the E-cadherin mRNA expression was decreased, whereas the mRNA levels of N-cadherin, α-SMA, and vimentin were upregulated in TGF-β1–induced A549 cells. Compared with the model group, the expression of E-cadherin was increased, and N-cadherin, α-SMA, and vimentin mRNA levels were downregulated after QM treatment at 125, 250, or 500 μg/ml (Figure 5B). Western blot analysis confirmed the qPCR results (Figures 5C,D). Together, these results demonstrated that QM inhibits EMT in TGF-β1–induced A549 cells.

### QM Facilitated ECM Degradation in TGF-β1–Induced A549 Cells

**FIGURE 6:**
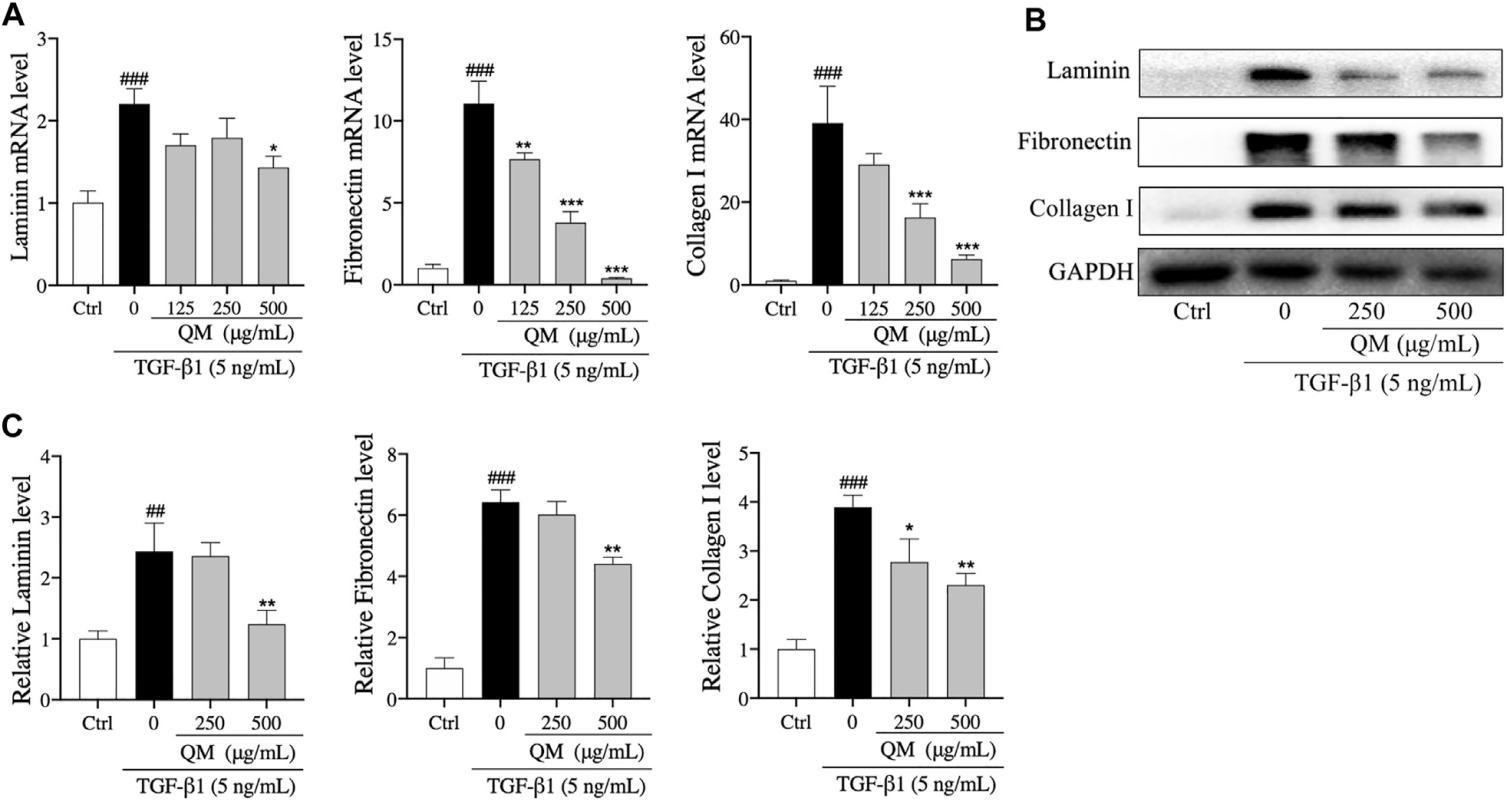
QM promoted the degradation of ECM in TGF-β1–induced A549 cells. **(A)** After incubation with TGF-β1 and/or QM for 72 h, qPCR analysis was performed and the relative mRNA levels of laminin, fibronectin, and collagen I in A549 cells were calculated and normalized to GAPDH. **(B,C)** The protein levels of laminin, fibronectin, and collagen I in A549 cells treated with TGF-β1 and/or QM for 72 h were determined by Western blot analysis. GAPDH was used as the loading control. Data are from three independent experiments. ^##^ *p* < 0.01, ^###^ *p* < 0.001 versus the control group; *p < 0.05, **p < 0.01, and ***p < 0.001 versus the model group (TGF-β1, 5 ng/ml).

The above findings revealed that EMT occurred in A549 cells after induction with TGF-β1. Cells with features of mesenchymal cells, such as fibroblasts and myofibroblasts, could secrete ECM proteins, such as collagen I, FN, and LN (Thiery et al., 2009). Excessive deposition of ECM is an important cause of organ fibrosis (Sun et al., 2013). In addition, the results of Reactome pathway enrichment from network pharmacology showed that QM could prevent PF *via* degrading ECM (Figure 3). To investigate the mechanisms by which QM stimulates ECM degradation, qPCR and Western blot analyses were conducted to investigate the protein and mRNA expression levels of key ECM markers, including LN, FN, and collagen I. The expression of collagen I, FN, and LN was significantly upregulated at the mRNA level in TGF-β1–induced A549 cells. QM treatment for 72 h decreased the level of collagen I, FN, and LN in a dose-dependent manner (Figure 6A). At the protein level, QM significantly reduced the TGF-β1–induced A549 cells which resulted in the increase in LN, FN, and collagen I levels. The inhibitory effects of QM on ECM accumulation were the strongest at a concentration of 500 μg/ml (Figures 6B,C). Together, these results suggested that QM stimulates ECM degradation in TGF-β1–induced A549 cells.

### QM Inhibited the TGF-β/Smad3 Pathway

**FIGURE 7:**
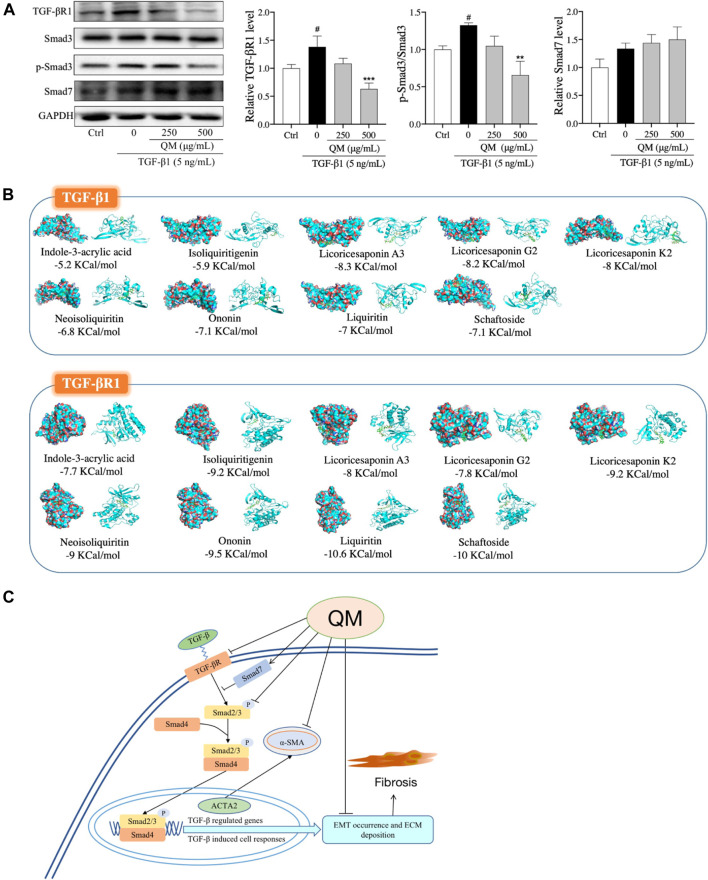
QM inhibited the TGF-β1/Smad3 pathway to block PF progression. **(A)** After incubation with QM and TGF-β1 for 72 h, the protein levels of TGF-βR1, Smad3, *p*-Smad3 (S423/S425), and Smad7 in A549 cells were determined by Western blot analysis. GAPDH was used as the loading control. The ratio of *p*-Smad3/Smad3 was calculated from three independent experiments after normalization to GAPDH. ^#^ *p* < 0.05 versus the control group; **p < 0.01 and ***p < 0.001 versus the model group (TGF-β1 5 ng/ml). **(B)** The molecular docking simulation results, including the compounds, the targets, and the lowest binding free energy. Green rectangles represent the compounds. The diagrams on the left are the surface appearances and those on the right are cartoon appearances of targets. **(C)** Scheme demonstrating the molecular mechanism underlying the effects of QM in preventing and treating PF.

TGF-β/Smads signaling is a classical pathway in the occurrence and progression of organ fibrosis (He X. et al., 2020), giving rise to the occurrence of EMT and ECM deposition (Pardali et al., 2017). Moreover, the results of our network pharmacological analysis also showed that QM may exert its anti-PF effects through the TGF-β signaling pathway (Figures 3B–D). To further verify whether QM could regulate the TGF-β/Smads pathway, TGF-βR1, Smad7, and Smad3 and its phosphorylation, as downstream proteins of TGF-β1, were detected by western blot and/or qPCR. As shown in Figure 7A; Supplementary Figure S2, TGF-βR1 was significantly upregulated, and Smad3 phosphorylation was induced by TGF-β1 in the model group. After QM treatment for 72 h, these indicators were downregulated. Moreover, in TGF-β1–stimulated A549 cells, there was no change in the Smad3 protein level in the QM-treated group, which meant that QM inhibited the TGF-β1–induced increase in the p-Smad3/Smad3 ratio. In addition, as an inhibitor of the TGF-β/Smad3 pathway, TGF-β1 could increase the expression of Smad7 due to feedback action (Wang et al., 2014). But QM treatment had no effect on the increased Smad7 level induced by TGF-β1. Taken together, these results indicated that QM could inhibit the TGF-β1/Smad3 pathway in TGF-β1–induced A549 cells.

### Analysis of Effective QM Components in the Rat Plasma

To identify the effective components of QM against PF, the QM components that could be absorbed into the bloodstream were characterized by UHPLC/IM-QTOF-MS. By comparing the MS data, the peaks that appeared in the corresponding positions of the QM-administered plasma and QM extract but did not appear in the blank rat plasma were considered as the prototype compounds absorbed into the plasma of rats. Accordingly, 10 prototype compounds were identified in the rat plasma: indole-3-acrylic acid from glehniae radix (Bei-sha-shen), schaftoside from pinelliae rhizoma (Ban-xia), and ononin from astragali radix (Huang-qi), and other seven components were from glycyrrhizae radix et rhizoma (Gan-cao), such as liquiritin, isoliquiritigenin, neoisoliquiritin, licoricesaponin A3, licoricesaponin G2, yunganoside G1, and licoricesaponin K2. The detailed information expressed by # is displayed in Supplementary Table S1. These results demonstrated that glycyrrhizae radix et rhizoma (Gan-cao) might be a key botanical drug of QM for preventing PF. However, the real and potential compounds responsible for the anti-PF effect of QM should be further identified in the mouse model with PF.

### Molecular Docking

We further explored the relationship between i) the nine effective components that can be absorbed into the bloodstream and ii) the TGF-β signaling pathway. Molecular docking was used to analyze the binding energies of the nine compounds with TGF-β1 and TGF-βR1. As shown in Figure 7B, the nine compounds all had good binding affinities with TGF-β1 and TGF-βR1, especially the compounds from glycyrrhizae radix et rhizome (Gan-cao), such as liquiritin, isoliquiritigenin, neoisoliquiritin, licoricesaponin A3, licoricesaponin G2, and licoricesaponin K2. The molecular docking results indicated that the nine compounds from QM could all potentially regulate the TGF-β signaling pathway. However, the pharmacology action of these active components in inhibiting the TGF-β signaling pathway needs to further investigate in the cell or animal models.

## Discussion

In our study, UHPLC/Q-TOF-MS analysis was first used to analyze the composition of QM, leading to the identification of 56 components. Combined with the PubChem database and the literature studies, 43 compounds were identified or tentatively characterized. Based on these components, network pharmacology was used to predict the potential mechanisms of QM in preventing PF. First, 452 potential therapeutic targets of QM were obtained through several databases and the literature studies, and a network of 43 ingredients and 452 targets was established, from which the multicomponent and multi-target characteristics of the TCM formula were observed. Meanwhile, 643 genes related to PF were screened, and 70 overlapping targets between QM and PF were identified. Based on the STRING database and 70 overlapping targets, a PPI network was established and analyzed, from which the main targets of QM in anti-PF function were obtained, including TNF, MMPs, MAPKs, SRC, CASP3, AKT1, EGFR, IL2, SMAD3, TGFB1/2, and TGFBR1. These targets participate in many progresses involved in PF, such as the inflammatory response, ECM deposition, EMT, and fibroblast differentiation (Hou et al., 2018; Espindola et al., 2021). In addition, a previous study screening drugs by scoring viral fibrosis based on MAPK activity indicated EGFR is a main regulator of COVID-19–related fibrosis (Vagapova et al., 2021). Our GO functional enrichment results showed that QM mainly influences ECM degradation, mesenchymal cell differentiation, EMT, protein tyrosine and kinase activity, TGF-β and type II TGF-β receptor binding, fibroblast growth factor–activated receptor activity, and fibroblast growth factor binding, which are closely connected with the progression of PF. The KEGG and Reactome pathway enrichment analyses mainly revealed the involvement of the FoxO, Rap1, MAPK, VEGF, and TGF-β signaling pathways, activation of MMPs, and degradation of the ECM and collagen. Taken together, the results of network pharmacology indicated that EMT, fibroblast activation, ECM degradation, and the TGF-β/Smads signaling pathway are key mechanisms underlying the anti-PF effects of QM, among which the EMT and ECM accumulation are downstream actions of the TGF-β/Smads signaling pathway (Xu et al., 2009; Lederer and Martinez, 2018; Chen, 2020). However, except for the TGF-β pathway, the potential roles of other signaling pathways as targets of QM should be further investigated, based on our network pharmacology results.

To verify the effects of QM and the mechanism underlying TGF-β/Smads signaling pathway regulation, ECM degradation, and EMT inhibition, TGF-β1–induced A549 cells were used to conduct a series of experiments. The TGF-β/Smads signaling pathway was shown to mediate EMT (Pardali et al., 2017; Yao et al., 2019). Notably, lung cancer cells infected by SARS-CoV-2 could induce metabolic and transcriptional changes consistent with EMT (Stewart et al., 2021). In addition, TGF-β–induced fibroblast activation and myofibroblast differentiation is a central pathway of PF, which could increase ECM production and abnormal deposition. ECM deposition is a key factor in the development of tissue remodeling and may lead to impaired lung function and symptoms of diseases such as asthma, COPD, and IPF (Liu et al., 2021). Moreover, previous studies have shown that the level of TGF-β is elevated in COVID-19 patients’ serum and upper airway samples (Montalvo Villalba et al., 2020; Ghazavi et al., 2021). Therefore, excessive ECM deposition is a main trigger for PF formation in COVID-19 convalescent patients (Habermann et al., 2020). In the present study, we first measured the markers related to EMT and ECM accumulation to evaluate the effects of QM in attenuating EMT and promoting ECM degradation. Then, the TGF-β1/Smad3 pathway was evaluated to confirm the mechanisms underlying the effects of QM. As shown in Figure 7C, our results demonstrate that QM can block the TGF-β1/Smad3 pathway to inhibit EMT and ECM deposition, which might be a critical functional mechanism of QM against PF.

To further analyze the possible active components of QM, the plasma of rats administrated with QM was analyzed by UHPLC/IM-QTOF-MS, and 10 active ingredients were obtained, among which the seven are originated from glycyrrhizae radix et rhizome (Gan-cao). Previous studies have shown that the components of glycyrrhizae radix et rhizome (Gan-cao) such as liquiritin, isoliquiritigenin, isoangustone A, and glycyrrhizin, could prevent organ fibrosis *via* many pathways, such as the TGF-β/Smad3, NF-κB, and MAPK signaling pathways (Li et al., 2010; Guan et al., 2012; Yan et al., 2018). Moreover, the ononin that originated from astragali radix (Huang-qi) was proven to inhibit cardiac fibrosis *via* the AMPK/mTOR signaling pathway (Pan et al., 2021). In addition, the binding energies were calculated based on molecular docking simulations, from which we found that the nine components potentially own the ability to bind to TGF-β1 and TGF-βR1 and, hence, regulate the TGF-β signaling pathway.

## Conclusion

In summary, we identified 56 chemical compounds from the QM extract and 10 effective components from the plasma of QM-administrated rats, based on which, we conducted a network pharmacology analysis predicting that TGF-β–mediated EMT inhibition and ECM degradation are potential mechanisms underlying the anti-PF effects of QM. Furthermore, QM is proven to attenuate EMT and degrade ECM *via* the inhibition of the TGF-β1/Smad3 signaling pathway in TGF-β1–induced A549 cells. These findings shed light on the molecular mechanism and the possible active components of QM underlying its anti-PF effects, thus supporting the clinical application of QM for COVID-19 convalescent patients.

## Data Availability

The original contributions presented in the study are included in the article/Supplementary Material; further inquiries can be directed to the corresponding authors.
