# Supplementary material for: Network Pharmacology and Experimental Assessment to Explore the Pharmacological Mechanism of Qimai Feiluoping Decoction Against Pulmonary Fibrosis
Source: Front Pharmacol. 2021 Dec 3;12:770197. doi: 10.3389/fphar.2021.770197 (PMC8678473; doi:10.3389/fphar.2021.770197)
Supplement: Supplementary file 7 [file DataSheet1.docx]

**Network pharmacology and experimental validation to explore the pharmacological mechanism of Qimai Feiluoping Decoction against pulmonary fibrosis**

Yingying Yang^1,2*^, Lu Ding^3*^, Tingting Bao^1,2*^, Yaxin Li^4^, Jing Ma^5^, Qingwei Li^2^, Zezheng Gao^2^, Siyu Song^4^, Jing Wang^5^, Jiachao Zhao^4^, Ziyuan Wang^5^, Daqing Zhao^3^, Xiangyan Li^3^, Zeyu Wang^6#^, Linhua Zhao^2#^; Xiaolin Tong^2#^

^1^Graduate College, Beijing University of Chinese Medicine, Beijing 100029, China.

^2^Institute of Metabolic Diseases, Guang’anmen Hospital, China Academy of Chinese Medical Sciences, Beijing 100053, China.

^3^Jilin Ginseng Academy, Key Laboratory of Active Substances and Biological Mechanisms of Ginseng Efficacy, Ministry of Education, Jilin Provincial Key Laboratory of Bio-Macromolecules of Chinese Medicine, Changchun University of Chinese Medicine, Changchun 130017, China.

^4^College of Integrated Traditional Chinese and Western Medicine, Changchun University of Chinese Medicine, Changchun 130017, China.

^5^Affiliated Hospital of Changchun University of Chinese Medicine, Changchun 130021, China.

^6^Department of Scientific Research, Changchun University of Chinese Medicine, Changchun 130017, China.

* These authors contributed equally to this work.

#**Corresponding authors:** Zeyu Wang, Department of Scientific Research, Changchun University of Chinese Medicine, 1035 Boshuo Road, Changchun, Jilin, 130017 China. Email: [zeyu781022@163.com](mailto:zeyu781022@163.com). Linhua Zhao, Molecular Biology Laboratory of Guang’anmen Hospital, China Academy of Chinese Medical Sciences, Beijing 100053, China. E-mail: [melonzhao@163.com](mailto:melonzhao@163.com). Xiaolin Tong, Department of Endocrinology of Guang’anmen Hospital, China Academy of Chinese Medical Sciences, Beijing 100053, China. E-mail: [tongxiaolin@vip.163.com](mailto:tongxiaolin@vip.163.com).

Table S1. Retention time (RT), accurate mass, fragment ions, predicted chemical formula, mass error (PPM), source and identification of active components in QM were detected by UHPLC/Q-TOF-MS.

| **NO.** | **RT  (min)** | **Observed  m/z** | **Adducts** | **Formula** | **Mass error (ppm)** | **MS/MS** | **Identified compounds** | **Source** |
| --- | --- | --- | --- | --- | --- | --- | --- | --- |
| 1 | 3.07 | 577.1346 | [M+H]^+^ | C_30_H_24_O_12_ | 1.04 | 403.0828, 328.1377, 310.1272, 166.0857 | Proanthocyanidin A1 or isomer | Bai-zhu/Bei-sha-shen |
| 2 | 3.44 | 315.0724 | [M–H]^–^ | C_13_H_16_O_9_ | 0.63 | 304.9113, 235.9252, 206.9718, 145.9305, 135.9014, 125.8726 | 1,5-anhydro-6-O-(3,4,5-trihydroxybenzoyl)-D-glucitol or isomer | Chen- pi |
| 3 | 3.56 | 197.0459 | [M–H]^–^ | C_9_H_10_O_5_ | 1.51 | 197.0455, 191.0186, 179.0340, 151.0400 | Danshensu or isomer | Dan-shen |
| 4 | 3.68 | 329.0893 | [M–H]^–^ | C_14_H_18_O_9_ | 0.42 | 211.0243, 167.0350, 119.0495, 108.0211 | 1-O-vanillin-glucoside or isomer | Nan-sha-shen/Bei-sha-shen |
| 5 | 3.73 | 254.1384 | [M+H]^+^ | C_13_H_19_NO_4_ | –0.79 | 254.1377, 147.0432, 115.0538 | Codonopsinol B or isomer | Dang-shen |
| 6 | 3.76 | 431.119 | [M–H]^–^ | C_18_H_24_O_12_ | –0.14 | 431.1180, 359.0973, 216.9808, 137.0241 | Asperulosidic acid or isomer | Huang-qi |
| 7 | 3.94 | 268.1541 | [M+H]^+^ | C_14_H_21_NO_4_ | –0.75 | 268.1534, 180.0871, 121.0646, 115.0640 | Codonopsine or isomer | Dang-shen |
| 8 | 4.17 | 253.1289 | [M+H]^+^ | C_10_H_20_O_7_ | 3.17 | 253.1286, 180.9571, 152.0585, 120.0804 | 14-Hydroxy-3,6,9,12-tetraoxatetradecan-1-oic acid or isomer | Bai-zhu |
| 9 | 4.73 | 385.0787 | [M–H]^–^ | C_16_H_18_O_11_ | 0.26 | 385.0762, 321.0275, 299.0767, 191.0192 | Methoxy-trihydroxycoumarin hexoside or isomer | Chen-pi |
| 10# | 4.97 | 188.0696 | [M+H]^+^ | C_11_H_9_NO_2_ | –4.81 | 159.0912, 146.0595, 130.0646, 115.0541 | Indole-3-acrylic acid or isomer | Bei-sha-shen |
| 11 | 5 | 232.1178 | [M–H]^–^ | C_10_H_19_NO_5_ | –0.56 | 232.1192, 203.0821, 152.0119, 108.0212 | Boc-O-methyl-L-threonine or isomer | Zhe-bei-mu |
| 12 | 5.24 | 351.1277 | [M–H]^–^ | C_14_H_24_O_10_ | –0.57 | 351.1293, 291.0990, 207.0881 | Diacetin or isomer | Zhe-bei-mu |
| 13 | 5.49 | 165.0561 | [M–H]^–^ | C_9_H_10_O_3_ | 0.18 | 165.0559, 163.0394, 119.0499, 99.9253 | 3-(4-Hydroxyphenyl) propionic acid or isomer | Gan-cao |
| 14^a^ | 5.64 | 493.228 | [M+FA]^–^ | C_21_H_36_O_10_ | –2.45 | 325.0077, 313.0562, 235.9252, 146.9632, 112.9852 | Atractyloside A | Bai-zhu |
| 15 | 5.72 | 385.0786 | [M–H]^–^ | C_16_H_18_O_11_ | 2.33 | 385.0769, 313, 0563, 191.0189, 116.9279 | Methoxy-trihydroxycoumarin hexoside or isomer | Chen-pi |
| 16 | 6.1 | 385.0746 | [M–H]^–^ | C_16_H_18_O_11_ | –0.80 | 385.0770, 367.0659, 191.0193, 116.9281 | Methoxy-trihydroxycoumarin hexoside or isomer | Chen-pi |
| 17 | 6.63 | 239.0551 | [M–H]^–^ | C_11_H_12_O_6_ | –0.46 | 239.0560, 179.0345, 167.0346, 135.0438, 119.0499 | 2-Succinyl-6-hydroxycyclohexa-2,4-diene-1-carboxylic acid or isomer | Shan-zha |
| 18 | 6.77 | 179.0349 | [M–H]^–^ | C_9_H_8_O_4_ | –0.11 | 179.0340, 149.0240, 135.0449, 133.0291, 105.0344 | Caffeic Acid or isomer | Dan-shen |
| 19 | 6.9 | 385.0745 | [M–H]^–^ | C_16_H_18_O_11_ | –0.83 | 385.0771, 367.0664, 191.0192, 116.9280 | Methoxy-trihydroxycoumarin hexoside or isomer | Chen-pi |
| 20 | 7.07 | 417.1171 | [M–H]^–^ | C_21_H_22_O_9_ | –0.50 | 417.1177, 269.0606, 255.0660, 135.0082, 119.0498 | Liquiritin or isomer | Gan-cao |
| 21 | 7.62 | 431.1919 | [M–H]^–^ | C_20_H_32_O_10_ | –0.09 | 421.1625, 383.1214, 329.0310, 167.0372 | 5,6,7, 8-tetraethyl dodecoate or isomer | Chen-pi |
| 22 | 7.69 | 593.1509 | [M–H]^–^ | C_27_H_30_O_15_ | –0.07 | 593.1505, 383.0767, 353.0663, 297.0767 | Lonicerin or isomer | Chen-pi |
| 23^a^ | 7.99 | 623.1615 | [M–H]^–^ | C_28_H_32_O_16_ | –0.05 | 623.1645, 413.0877, 383.0772, 312.0637, 289.0717 | Complanatuside | Huang-qi |
| 24 | 8.24 | 433.1117 | [M–H]^–^ | C_21_H_22_O_10_ | –0.55 | 433.1142, 271.0621, 151.0038 | Naringenin-7-O-glucoside or isomer | Chen-pi |
| 25^a^# | 8.44 | 563.1396 | [M–H]^–^ | C_26_H_28_O_14_ | –0.19 | 563.1392, 371.0975, 255.0666, 165.0556 | Schaftoside | Ban-xia |
| 26^a^ | 9.56 | 491.1155 | [M+FA]^–^ | C_22_H_22_O_10_ | –0.92 | 283.0611, 255.0664, 211.0395, 135.0085 | Calycosin 7-O-glucoside | Huang-qi |
| 27^a^# | 9.83 | 417.1172 | [M–H]^–^ | C_21_H_22_O_9_ | –0.48 | 417.1186, 255.0668, 119.0504 | Liquiritin | Gan-cao |
| 28^a^# | 9.83 | 257.0808 | [M+H]^+^ | C_15_H_12_O_4_ | 0 | 257.0806, 249.0546, 165.0690, 137.0230 | Isoliquiritigenin | Gan-cao |
| 29 | 11.02 | 537.1021 | [M–H]^–^ | C_27_H_22_O_12_ | –3.35 | 537.1044, 339, 0510, 315.0502, 185.0241, 109.0288 | Lithospermic acid or isomer | Dan-shen |
| 30 | 11.12 | 581.1882 | [M+H]^+^ | C_27_H_32_O_14_ | 3.1 | 581.1854, 347.0749, 273.0753, 153.0178 | Narirutin or isomer | Chen-pi |
| 31^a^ | 11.13 | 579.1729 | [M–H]^–^ | C_27_H_32_O_14_ | 0.16 | 579.1718, 271.0618, 151.0039 | Naringin | Gan-cao |
| 32 | 11.94 | 283.0611 | [M–H]^–^ | C_16_H_12_O_5_ | –0.07 | 283.0612, 171.9464, 119.0500 | Calycosin or isomer | Huang-qi |
| 33^a^ | 11.94 | 432.3454 | [M+H]^+^ | C_27_H_45_NO_3_ | –3.94 | 285.0748, 253.0481, 137.0228 | Peimine | Zhe-bei-mu |
| 34^a^ | 12.08 | 609.1887 | [M–H]^–^ | C_28_H_34_O_15_ | 1 | 609.1831, 301.0731, 201.0196, 151.0040 | Hesperidin | Chen-pi |
| 35^a^ | 12.31 | 609.1835 | [M–H]^–^ | C_28_H_34_O_15_ | 0.15 | 609.1834, 323.0532, 301.0729 | Neohesperidin | Chen-pi |
| 36^a^ | 12.66 | 430.3307 | [M+H]^+^ | C_27_H_43_NO_3_ | –1.86 | 430.3294, 412.3210, 396.2887, 176.1430 | Peiminine | Zhe-bei-mu |
| 37 | 12.77 | 493.1147 | [M–H]^–^ | C_26_H_22_O_10_ | 1.21 | 493.1127, 295.0604, 185.0242, 109.0291 | Salvianolic acid A or isomer | Dan-shen |
| 38^a^ | 13.24 | 441.1741 | [M+FA]^–^ | C_20_H_28_O_8_ | –0.66 | 389.0843, 171.9463, 116.9280 | Lobetyolin | Dang-shen |
| 39 | 13.4 | 693.2762 | [M–H]^–^ | C_34_H_46_O_15_ | –0.04 | 693.2754, 565.2645, 341.1049, 171.9465, 116.9280 | Nomilin glucoside or isomer | Chen-pi |
| 40^a^# | 13.83 | 417.1178 | [M–H]^–^ | C_21_H_22_O_9_ | –0.33 | 417.1184, 255.0662, 135.0084, 119.0500 | Neoisoliquiritin | Gan-cao |
| 41^a^# | 14.07 | 475.123 | [M+FA]^–^ | C_22_H_22_O_9_ | –0.40 | 321.0412, 295.0616, 293.0462, 109.0295 | Ononin | Huang-qi |
| 42 | 14.33 | 717.1445 | [M–H]^–^ | C_36_H_30_O_16_ | –2.37 | 493.1127, 295.0604, 185.0242, 109.0291 | Danfensuan B or isomer | Dan-shen |
| 43^a^ | 15.88 | 507.1478 | [M+FA]^–^ | C_23_H_26_O_10_ | –0.67 | 339.0507, 321.0408, 295.0614, 116.9282 | Methylnissolin-3-O-Glucoside | Huang-qi |
| 44^a^ | 16.03 | 283.0613 | [M–H]^–^ | C_16_H_12_O_5_ | 0 | 283.0610, 233.0854, 185.0242, 135.0447 | Calycosin | Huang-qi |
| 45 | 16.59 | 593.1905 | [M–H]^–^ | C_28_H_34_O_14_ | 4.71 | 593.1873, 285.0779, 243.0665, 116.9284 | Didymin or isomer | Chen-pi |
| 46^a^ | 17.43 | 271.0623 | [M–H]^–^ | C_15_H_12_O_5_ | 0.37 | 271.0608, 193.0346, 119.0500, 113.0240 | Naringenin | Gan-cao |
| 47# | 17.63 | 983.4501 | [M–H]^–^ | C_48_H_72_O_21_ | 0.07 | 983.4503, 821.3972, 803.3861, 645.3640, 351.0572, 193.0357 | Licoricesaponin A3 or isomer | Gan-cao |
| 48 | 17.86 | 723.2173 | [M–H]^–^ | C_33_H_40_O_18_ | 4.14 | 723.2133, 417.1190, 387.0718, 359.0771, 171.9465 | Ligustroflavone or isomer | Chen-pi |
| 49# | 18.72 | 837.3894 | [M–H]^–^ | C_42_H_62_O_17_ | –2.50 | 837.3927, 661.3598, 485.3273, 351.0575, 193.0356 | Licoricesaponin G2 or isomer | Gan-cao |
| 50# | 20.73 | 837.3901 | [M–H]^–^ | C_42_H_62_O_17_ | –1.67 | 837.3926, 661.3594, 485.3273, 351.0575, 193.0359 | Licoricesaponin G2 or isomer | Gan-cao |
| 51# | 21.72 | 985.4615 | [M–H]^–^ | C_48_H_74_O_21_ | –3.65 | 985.4655, 839.4050, 664.3727, 497.1138, 339.0925 | Yunganoside G1 or isomer | Gan-cao |
| 52# | 21.96 | 821.4063 | [M–H]^–^ | C_42_H_62_O_16_ | 1.18 | 821.3981, 645.3544, 351.0574, 193.0358 | Licoricesaponin K2 or isomer | Gan-cao |
| 53^a^ | 22.57 | 403.1376 | [M+H]^+^ | C_21_H_22_O_8_ | –2.74 | 403.1375, 301.0696, 183.0282, 149.0227 | Nobiletin | Chen-pi |
| 54# | 22.93 | 821.3945 | [M–H]^–^ | C_42_H_62_O_16_ | –0.26 | 821.3978, 645.3649, 497.1145, 351.0574, 289.0570 | Licoricesaponin K2 or isomer | Gan-cao |
| 55 | 23.42 | 433.1495 | [M+H]^+^ | C_22_H_24_O_9_ | 0.69 | 433.1490, 345.0594, 149.0229 | 3,3',4',5,6,7,8-Heptamethoxyflavone or isomer | Chen-pi |
| 56^a^ | 24.51 | 913.4851 | [M+FA]^–^ | C_45_H_72_O_16_ | 0.55 | 913.4811, 787.3568, 309.1743, 171.9463, 116.9283 | Isoastragaloside I | Huang-qi |

*^a^ The identification was certified by reference standards. # The complements were identified in rats’ serum contained QM.*

**Table S2.** Primer sequences for quantitative real-time PCR analysis

| **Gene name** | **Primer sequences** |
| --- | --- |
| Human N-Cadherin-F | ATCAAGCCTGTGGGAATCCG |
| Human N-Cadherin-R | AGCTGTGGGGTCATTGTCAG |
| Human E-Cadherin-F | CTGATGCTGATGCCCCCAATA |
| Human E-Cadherin-R | CTGCATCTTGCCAGGTCCTT |
| Human α-SMA-F | TAGCACCCAGCACCATGAAG |
| Human α-SMA-R | CTGCTGGAAGGTGGACAGAG |
| Human LN-F | GGACGAAAAGAAGTGCTTCCT |
| Human LN-R | GCAGGGATACCATTCTCTGACT |
| Human FN-F | ATGAGCTGCACATGTCTTGG |
| Human FN-R | TGGCACCGAGATATTCCTTC |
| Human Collagen I-F | TTCTGCAACATGGAGACTGG |
| Human Collagen I-R | AATCCATCGGTCATGCTCTC |
| Human Vimentin-F | GGACCAGCTAACCAACGACA |
| Human Vimentin-R | AAGGTCAAGACGTGCCAGAG |
| Human Smad7-F | AGAGGCTGTGTTGCTGTGAAT |
| Human Smad7-R | TCGCAGAGTCGGCTAAGGT |
| Human TGFBR1-F | ACGGCGTTACAGTGTTTCTG |
| Human TGFBR1-R | GCACATACAAACGGCCTATCTC |
| Human GAPDH-F | CACCCACTCCTCCACCTTTG |
| Human GAPDH-R | CCACCACCCTGTTGCTGTAG |

**Table S3.** The overlapping targets between QM and PF

| **NO.** | **Target** | **NO.** | **Target** | **NO.** | **Target** | **NO.** | **Target** | **NO.** | **Target** |
| --- | --- | --- | --- | --- | --- | --- | --- | --- | --- |
| 1 | ABCC1 | 15 | CHIT1 | 29 | IL2 | 43 | MMP2 | 57 | REN |
| 2 | ACE | 16 | CTSG | 30 | JAK2 | 44 | MMP3 | 58 | RNASE3 |
| 3 | ACE2 | 17 | EGFR | 31 | KDR | 45 | MMP7 | 59 | S100A9 |
| 4 | AKT1 | 18 | ELANE | 32 | KIT | 46 | MMP8 | 60 | SELE |
| 5 | ALB | 19 | F2 | 33 | LGALS3 | 47 | MMP9 | 61 | SELP |
| 6 | ALOX5 | 20 | FGFR1 | 34 | LTF | 48 | NOS2 | 62 | SERPINA1 |
| 7 | ANXA5 | 21 | FGFR2 | 35 | MAP2K1 | 49 | NOS3 | 63 | SMAD3 |
| 8 | BMP2 | 22 | FHIT | 36 | MAPK1 | 50 | NR1H4 | 64 | SRC |
| 9 | BMP7 | 23 | GSTM1 | 37 | MAPK14 | 51 | PDE5A | 65 | STAT1 |
| 10 | BRAF | 24 | GSTP1 | 38 | MAPK8 | 52 | PLG | 66 | TGFB1 |
| 11 | CASP3 | 25 | HMOX1 | 39 | MET | 53 | PPARG | 67 | TGFB2 |
| 12 | CAT | 26 | HRAS | 40 | MIF | 54 | PTPN11 | 68 | TGFBR1 |
| 13 | CCL5 | 27 | IGF1 | 41 | MMP1 | 55 | RAF1 | 69 | TNF |
| 14 | CDC42 | 28 | IGF1R | 42 | MMP12 | 56 | RARB | 70 | TEK |

**Table S4-S****9**. Detailed results of network pharmacological analysis. (Uploaded as separate Excel files).

Table S3. The targets of 43 active components of QM

Table S4. The terms of BP

Table S5. The terms of CC

Table S6. The terms of MF

Table S7. The terms of Reactome pathways

Table S8. The terms of KEEG pathways


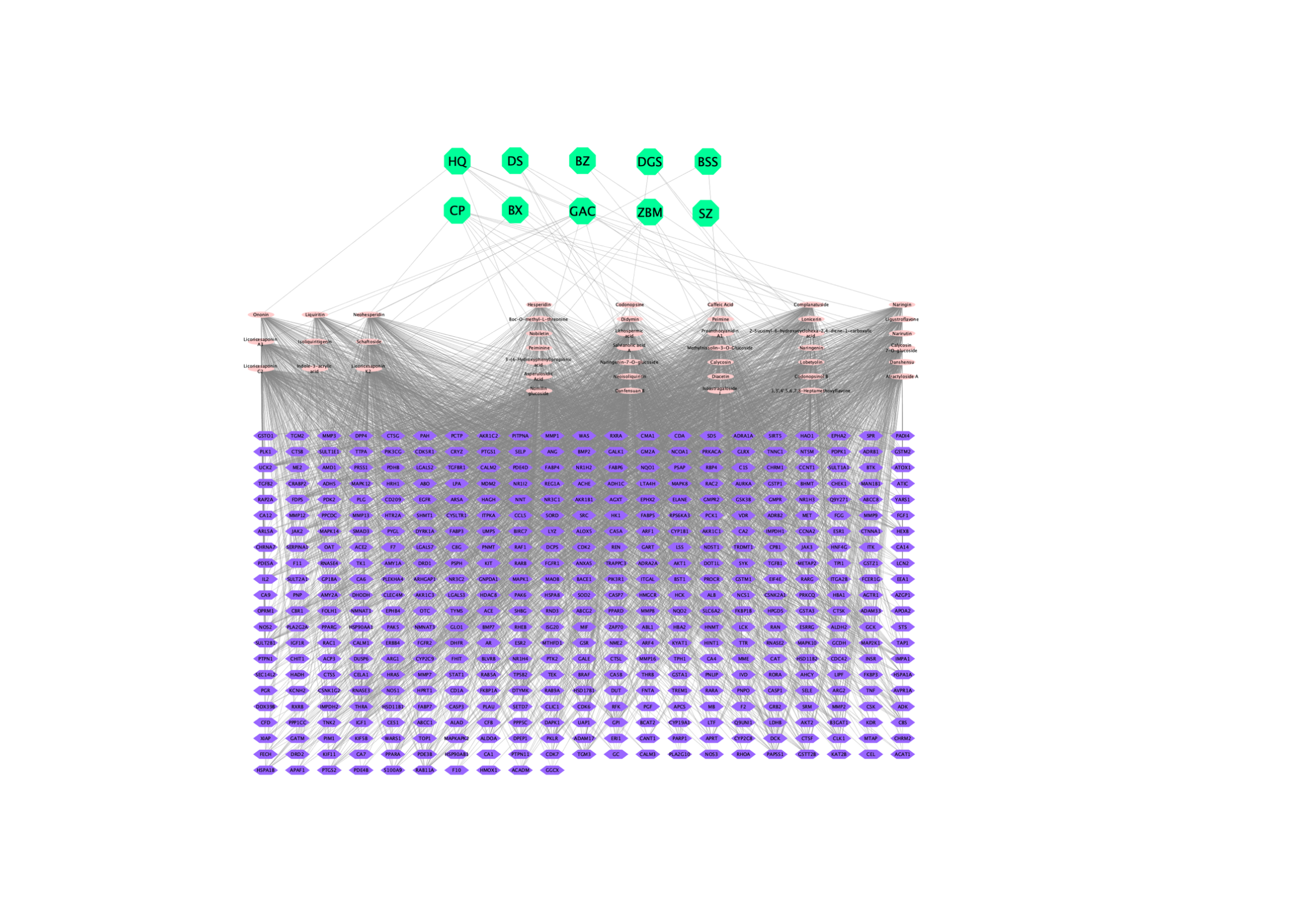


**Figure S1**. Construction of the network of 43 components–452 potential targets. The green octagon represents the sourcing herbs, the pink ellipse represents the 43 compositions, among which circled by the red box are plasma active ingredients, and the purple hexagon represents all potential targets of QM.


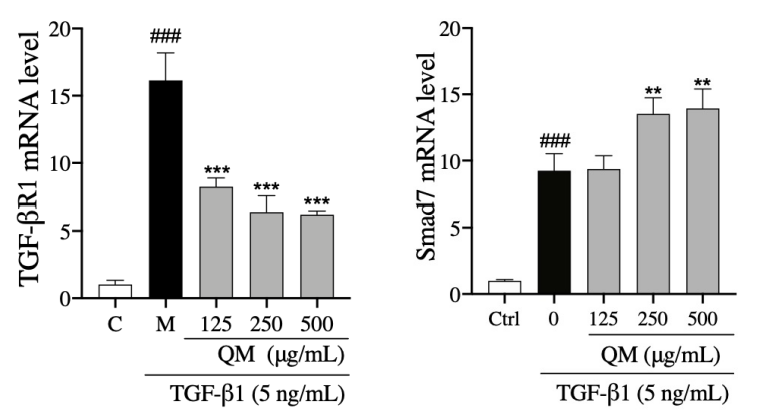


**Figure S2**. QM inhibited TGF-β1/Smad3 pathway to block PF progression. After QM and TGF-β1 incubation for 72 h, the mRNA levels of TGF-βR1, Smad7 in A549 cells were determined by qPCR analysis and normalized using GAPDH. ### *P* < 0.001 versus the Ctrl group. ***P* < 0.01 and ****P* < 0.001 versus the model group.
